# Fabrication of the Ni-NiCl_2_ Composite Cathode Material for Fast-Response Thermal Batteries

**DOI:** 10.3389/fchem.2021.679231

**Published:** 2021-05-17

**Authors:** Qianqiu Tian, Jiajun Wang, Wendi Xiang, Jun Zhao, Hao Guo, Jing Hu, Xiaopeng Han, Wenbin Hu

**Affiliations:** ^1^ School of Materials Science and Engineering, Tianjin University, Tianjin, China; ^2^ State Key Laboratory of Advanced Chemical Power Sources, Guizhou, China; ^3^ Shandong Engineering Research Center of Green and High-value Marine Fine Chemical, Weifang University of Science and Technology, Shouguang, China

**Keywords:** thermal battery, Ni-NiCl_2_ composite cathode, hydrogen reduction method, activation time, discharge ability

## Abstract

Thermal batteries with a high power density and rapid activation time are crucial for improving the fast response ability of sophisticated weapons. In this study, an Ni-NiCl_2_ composite was prepared *via* hydrogen reduction and employed as a cathode material. Discharge tests on a battery assembled using the fabricated composite revealed that its initial internal resistance decreased and the activation time reduced. Notably, the Ni-NiCl_2_ cathode increased the energy output by 47% (from 6.76 to 9.94 Wh in NiCl_2_ and Ni-NiCl_2_, respectively) with a cut-off voltage of 25 V; the power density of the novel battery system reached 11.4 kW/kg. The excellent performance of the thermal battery benefited from the high electrode potential and low internal resistance of Ni-NiCl_2_. This study contributes to the development of high-performance electrode materials for next-generation thermal battery-related technologies.

## Introduction

Thermally activated batteries (also called “thermal batteries”) are a kind of primary batteries, which apply molten salts as electrolytes and employ an internal pyrotechnic (heat) source to increase the temperature to operating conditions. Thermal batteries have been extensively used in weapons and emergency equipments (Guidotti et al., 2006; Masset and Guidotti, 2007). The activation time and power density significantly affect the response speed of weapon systems. Owing to their lower costs, adequate compatibility, and stable discharging properties, metal sulfides are regarded as ideal cathode materials for thermal batteries; examples of such metal sulfides include FeS_2_ (Au, 2003; Guidotti et al., 2006; Masset and Guidotti, 2008a; Liu et al., 2014; Choi et al., 2015), CoS_2_ (Masset and Guidotti, 2008b), NiS_2_ (Masset and Guidotti, 2008b) and polymetallic disulfide composites (Guidotti et al., 2006; Masset and Guidotti, 2008b; Hu et al., 2018b). Thermal batteries with a higher power density and rapid activation time are urgently required to keep up with the rapid development of weapon technologies (such as ejection seat in a fighter jet). A fast response is typically difficult to achieve using sulfide-based thermal batteries; moreover, the power density of such batteries rarely exceeds 10 kW/kg.

Compared to metal sulfides, NiCl_2_ cathodes possess various advantages, such as a higher output voltage and lower polarization resistance at a high current density, which makes them ideal replacements for sulfide cathodes. Moreover, the voltage of an NiCl_2_ cell can reach 2.55 V (Hu et al., 2017; Jin et al., 2017; Hu et al., 2018a), which is significantly higher than that of a disulfide cell (∼2 V). Additionally, their polarization resistance is lower at high current densities (Prakash et al., 2000; Hu et al., 2017). However, NiCl_2_ also has some drawbacks. First, the raw material (NiCl_2_ 6H_2_O) must be completely calcined to remove the crystal water content; these crystal water molecules can significantly decrease the safety and electrochemical performance of the cell. If the intrinsic crystal water is volatilized at a high temperature, it reacts with Li in the negative electrode to generate hydrogen; accordingly, a large amount of heat is released, which leads to the short-circuiting of the battery and even explosions. Second, a hydrolysis reaction simultaneously occurs during the removal of crystal water; this promotes the formation of nickel oxide, which exhibits poor conductivity and prolongs the activation time (Liu et al., 2017). Various strategies have been devoted to improving the performance of NiCl_2_-based cathode materials, including variable-temperature preparation (Liu et al., 2017), surface modification by carbon coating (Jin et al., 2017), and metal doping-based modification (Liu et al., 2014). However, the activation time still cannot meet the requirements necessary to ensure a fast response. Therefore, the development of high-performance NiCl_2_-based electrode materials is challenging (Wang et al., 2013; Hosseinifar and Petric, 2016; Rock et al., 2016; Gui et al., 2020).

The conductivity of materials is known to significantly affect the activation time of thermal batteries (Chu et al., 2016); a lower internal resistance reduces the activation during the early discharge process. In this study, an Ni-NiCl_2_ composite was designed and prepared *via* hydrogen reduction and the composite was found to exhibit excellent conductivity and a low polarization resistance. The Ni-NiCl_2_ cathode improved the output performance and significantly reduced the activation time, which decreased the initial internal resistance of the thermal battery. This study investigated an efficient cathode candidate and provides a useful path for developing novel hybrid materials for thermal batteries.

## Experimental Section

### Material Synthesis

**FIGURE 1:**
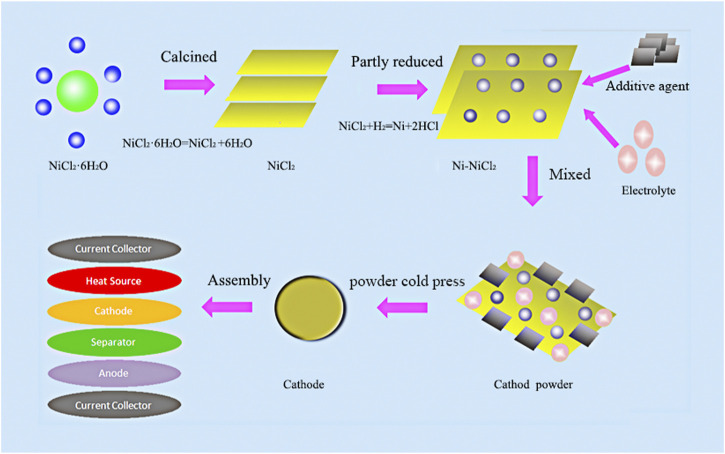
Schematic illustration of the preparation of Ni-NiCl_2_ composite cathode material and the assembly of the thermal battery.

NiCl_2_•6H_2_O (AR, Sinopharm Chemical Reagent Co., Ltd.) was first calcined at 250°Cfor 12 h to facilitate the removal of crystal water because the crystalline water content considerably decreases the performance and safety of thermal batteries (Chu et al., 2016; Liu et al., 2017). The sample was subsequently ground into powder and loaded in an Ar-filled quartz tube furnace. The temperature was increased to 750°C at a rate of 5°C/min and maintained for 1 h. Subsequently, the surface of the as-prepared sample was shaved off to get rid of the black surface impurities, after which the leftover golden sample represented calcined NiCl_2_. An Ni-NiCl_2_ sample was finally synthesized in the quartz tube furnace by reducing the calcined NiCl_2_ using an argon–hydrogen mixture (5 at% H_2_) at 300–450°C for 1 h. As a green and efficient method, hydrogen reduction can not only provide *in-situ* with high conductivity nickel, but also avoid the formation of other solid chemical components in the hydrogen process (Nogami et al., 2019). The chemical formula of each step during hydrogen reduction method is as follows: NiCl_2_•6H_2_O = = = NiCl_2_+6H_2_O, NiCl_2_+H_2_ = = = Ni+2HCl. The synthesis protocol is illustrated in Figure 1.

An all-Li electrolyte (9.6, 22, and 68.4 wt% of LiCl (AR, Sinopharm Chemical Reagent Co., Ltd.), LiF (AR, Sinopharm Chemical Reagent Co., Ltd.), and LiBr (AR, Sinopharm Chemical Reagent Co., Ltd.), respectively) was prepared after melting the components at 500°C (Masset and Guidotti, 2007). The cathode material was prepared by mixing Ni-NiCl_2_, the all-Li electrolyte, and an additive agent carbon in a mass ratio of 100:15:5 using a planetary ball mill at 800 rpm for 1 min. An Li-B alloy pellet (60 wt%Li, Grinm Group Co., Ltd.) was employed as the anode material (Guidotti and Masset, 2008). The separator was composed of 50 wt% of an LiCl-LiBr-LiF electrolyte and 50 wt% of MgO (AR, Sinopharm Chemical Reagent Co., Ltd.). The heating powder was prepared using Fe (≥ 98 wt%Fe, Grinm Group Co., Ltd.) and KClO_4_ (AR, Sinopharm Chemical Reagent Co., Ltd.) in a proportion of 84:16 wt%.

### Material Characterization

Thermogravimetry (TG) and differential scanning calorimetry (DSC) (TG-DSC;6300 Mettler Toledo) were employed to investigate the stability of the synthesized materials. Field emission scanning electron microscopy (FE-SEM; Hitachi S-4800, 30 kV) and transmission electron microscopy (TEM; JEOL JEM-2100F, 200 kV) were employed to investigate the structural properties. The crystal structures of the samples were characterized by X-ray diffraction (Bruker D8 Advanced, Cu Kα radiation).

### Single Cell Tests

A single cell was assembled using the Li-B alloy anode, separator based on the eutectic LiCl-LiBr-LiF, Ni-NiCl_2_ cathode, and Fe/KClO_4_ heating plate; The mass proportions of each component in the anode, electrolyte, and cathode are summarized in Supplementary Table S1. As a heat source, heating plate (Fe/KClO_4_) can make the internal temperature increased to ∼550°C (the temperature during the discharge test). The cell arrangement is as illustrated in Figure 1. The cathode, separator, and heating materials were pressed into sheets using a force of 1600 kN, resulting in corresponding diameters of 74 mm. The activation time and power density significantly affect the response speed of weapon systems. In order to ensure the credibility of the data and obtain the activation time, it is a reasonable method to use batteries tested instead of using single cells. In addition, single cell performance can be greatly affected by the environment. Based on these considerations, thirteen single cells were subsequently assembled into a thermal battery. The discharge test was conducted using a battery dedicated device (Supplementary Figure S1; Chengdu Oukai Technology), which was composed of a DC source, test load, voltage and current collector, and computer.

The parameters for the discharge tests were set as follows: a constant current density of 100 mA/cm^2^, loading a pulse current density (1 A/cm^2^, 10 ms) every 10 s, and a total of 10 pulses. The thermal battery was activated using an electrical signal, when DC source sent the electrical signal, the computer start to collect voltage and current value. When the thermal batteries received the electrical signal, the Fe/KClO_4_ heating plate would burn and the internal temperature would rapidly increase to ∼550°C, which made the LiCl-LiBr-LiF electrolyte melt. Then thermal batteries were activated and the data of voltage and current would be collected by computer system. The activation voltage was 25 V, which is 75% of the peak voltage in this work.

## Results and Discussion

**FIGURE 2:**
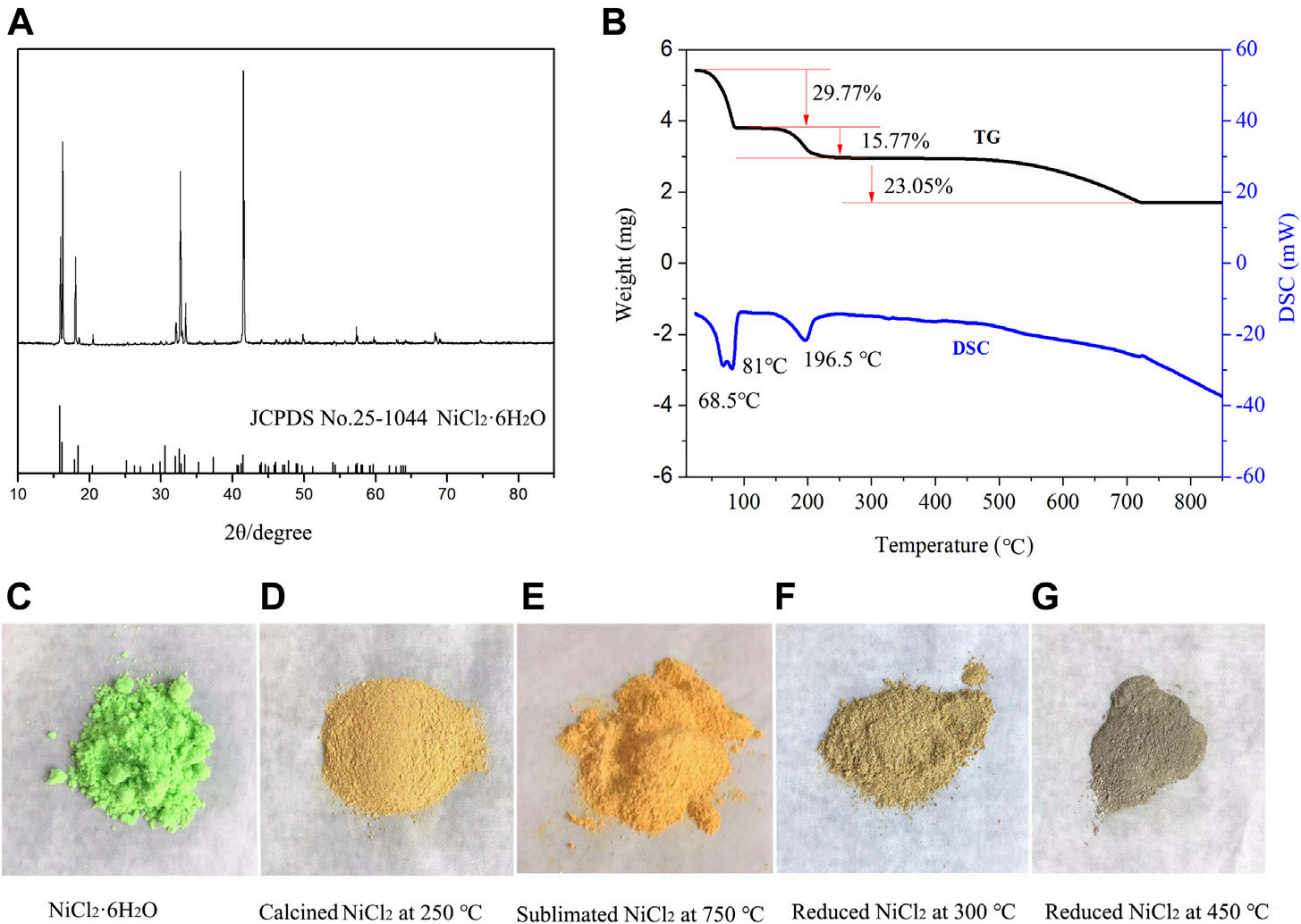
**(A)** XRD patterns of NiCl_2_.6H_2_O, **(B)** thermal analysis curves, and **(C)** pictures of NiCl_2_.6H_2_O, **(D)** NiCl_2_ calcinedat 250°C, **(E)** NiCl_2_ sublimated at 750°C, **(F)** NiCl_2_ reduced at 300°C, and **(G)** NiCl_2_ reduced at 450°C.

A previous report (Bette et al., 2015) indicated that four units of crystalline water are removed from NiCl_2_•6H_2_O at ∼80°C. However, it is difficult to remove completely the remaining two units even above 200°C, owing to the formation of a black nickel oxide that is produced *via* the hydrolysis reaction of NiCl_2_ with crystalline water (Liu et al., 2017). Figure 2A shows the characteristic peaks of NiCl_2_.6H_2_O (PDF card no. 25–1044) with a high purity. The TG curve in Figure 2B shows three noticeable weight loss areas with loss values of 29.77, 15.77, and 23.05%, respectively. Based on the theoretical chemical formula of NiCl_2_.6H_2_O, 3.93 and 1.95 units of crystal water are presumed to be lost at a temperature below 90°C and between 90 and 225°C, respectively. However, the third weight loss value (23.05%) does not correspond to the weight of the remaining 0.12 units of crystal water. This is because of the sublimation and pyrohydrolysis phenomena that appear when the treating temperature is above 500°C (Liu et al., 2017). The pyrohydrolysis products include black NiO compound and HCl gas are clearly observed in the calcining experiment (Supplementary Figure S2). Some studies have reported NiCl_2_•6H_2_O pyrohydrolysis products, such as Ni_2_Cl(OH)_3_, NiCl(OH), and NiCl_x_ (OH)_2-x_, which appears as an intermediate phase above 200°C (Bette et al., 2015; Bette et al., 2017). Additionally, hydrochloric acid is noted to be present in the gaseous product.

The DSC curve reveals three endothermic peaks (68.5, 81, and 196.5°C) and two endothermic regions (460–725 and above 725°C). Upon comparing the TG and DSC curves in the first stage, four units of crystal water are noted to be removed at temperatures below 90°C and the endothermic peak appears at 81°C; the peak at 68.5°C corresponds to the evaporation of free water. In the second stage, two additional units of crystal water are lost before the temperature reaches 250°C, and an endothermic peak appears at 195.5°C. In the third stage, pyrohydrolysis occurs at temperatures above 460°C, and the products include hydrochloric NiO impurities and HCl gas (Liu et al., 2017). In the last stage, nickel chloride particles transform into a layered structure. Notably, the material color changes from grass green to yellow and finally golden yellow throughout the calcining process (Figures 2C–E). When the calcined material is reduced by hydrogen at 300–450°C, metallic nickel appears, and the color gradually transforms into gray (Figures 2F,G).

**FIGURE 3:**
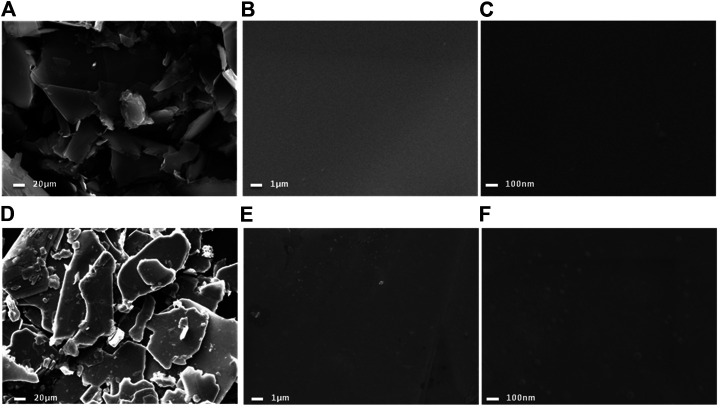
SEM images of **(A–C)** calcinedNiCl_2_ and **(D–F)** reduced Ni-NiCl_2_.

The calcined NiCl_2_ and reduced NiCl_2_ at 300°C (named Ni-NiCl_2_) were subsequently analyzed by SEM. As shown in Figures 3A,D, both the nickel chloride samples exhibit a typical layered structure with a size lesser than 80 mesh (∼a maximum size of 187.5 µm). However, the edges in Ni-NiCl_2_ are smoother and more rounded, compared to those in NiCl_2_. Moreover, the enlarged SEM images (Figures 3B,C,E,F) reveal the clean surface of calcinedNiCl_2_, and numerous particles on the surface of Ni-NiCl_2_. Therefore, a portion of the NiCl_2_ is presumed to be reduced to metallic Ni by the hydrogen treatment; this is further confirmed by the XRD and TEM results described below.

**FIGURE 4:**
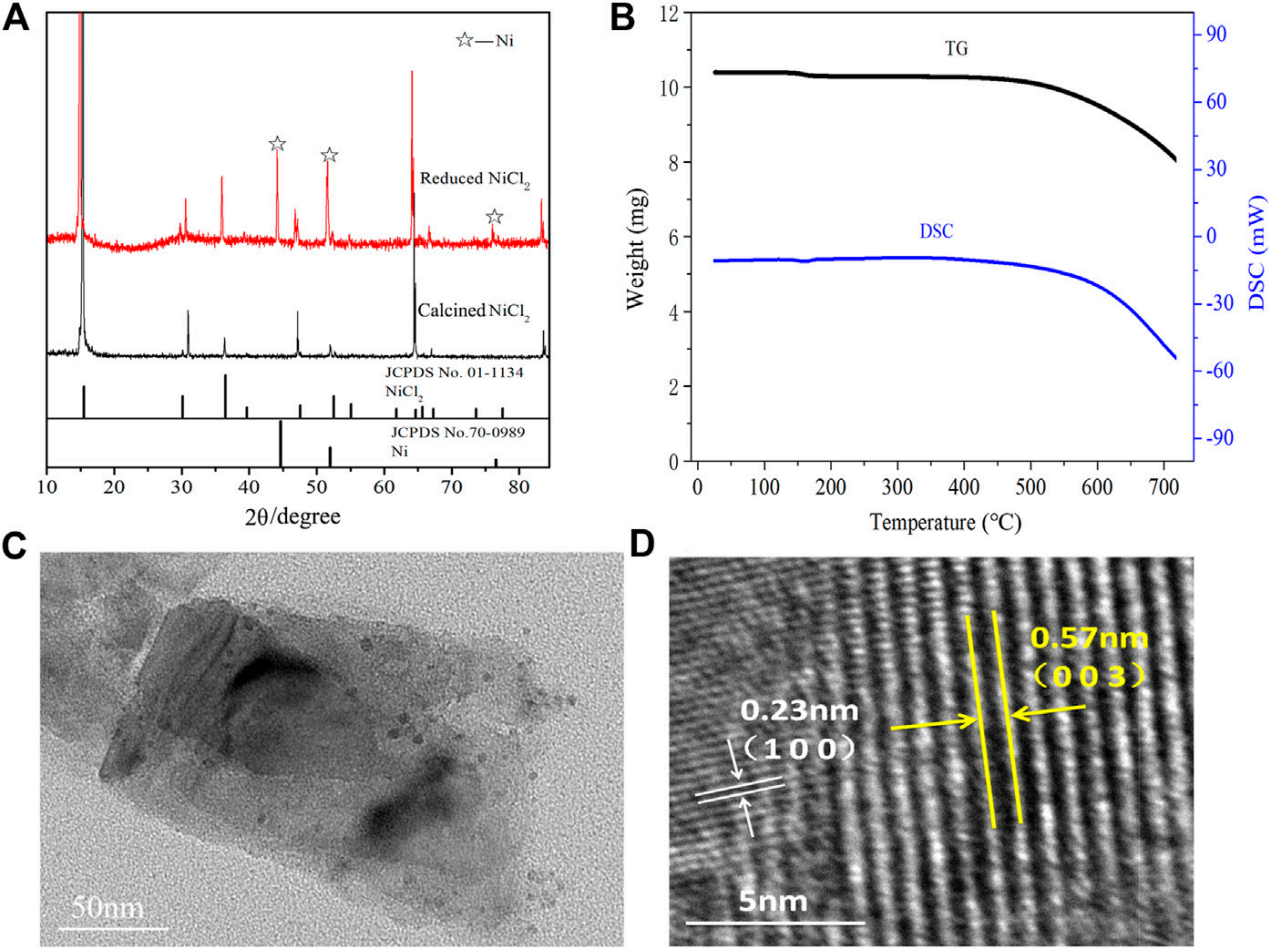
**(A)** XRD patterns of calcinedand reduced NiCl_2_, **(B)** thermal analysis curves of calcinedNiCl_2_, and **(C)** TEM and **(D)** HRTEM images of the Ni-NiCl_2_ composite materials.

As shown in Figure 4A, the calcinedNiCl_2_ exhibits characteristic peaks corresponding to anhydrous NiCl_2_ (PDF card no. 01–1134) after the high temperature treatment, indicating a complete removal of the crystal water (Liu et al., 2017). In parallel, some metallic Ni (PDF card no. 70–0989) appears in the peaks of Ni-NiCl_2_, which suggests the reduction of NiCl_2_ to Ni after the hydrogen reduction. Moreover, the energy-dispersive X-ray spectroscopy (EDS) results reveal that the mass ratio of Ni in Ni-NiCl_2_ (49.4 wt%) increases by 4.4% compared to that in NiCl_2_ (45.0 wt%) (Supplementary Figure S3). This result suggests that a portion of the nickel chloride is reduced to metallic nickel *via* hydrogen treatment, which is consistent with the XRD results. Meanwhile, thermal analysis was carried out on the calcined NiCl_2_ to further determine the state of crystal water. Figure 4B shows no notable weight loss or heat absorption/release peaks before 500°C, indicating the complete removal of water during sample preparation.

The microstructure of Ni-NiCl_2_ was further investigated by TEM. A sheet structure is visible in Figure 4C and solid nanoparticles with a size of ∼10 nm are embedded in the sheets. Figure 4D shows the (003) plane of NiCl_2_ and the (100) plane of metallic Ni, suggesting the co-existence of NiCl_2_ and metallic Ni.

**FIGURE 5:**
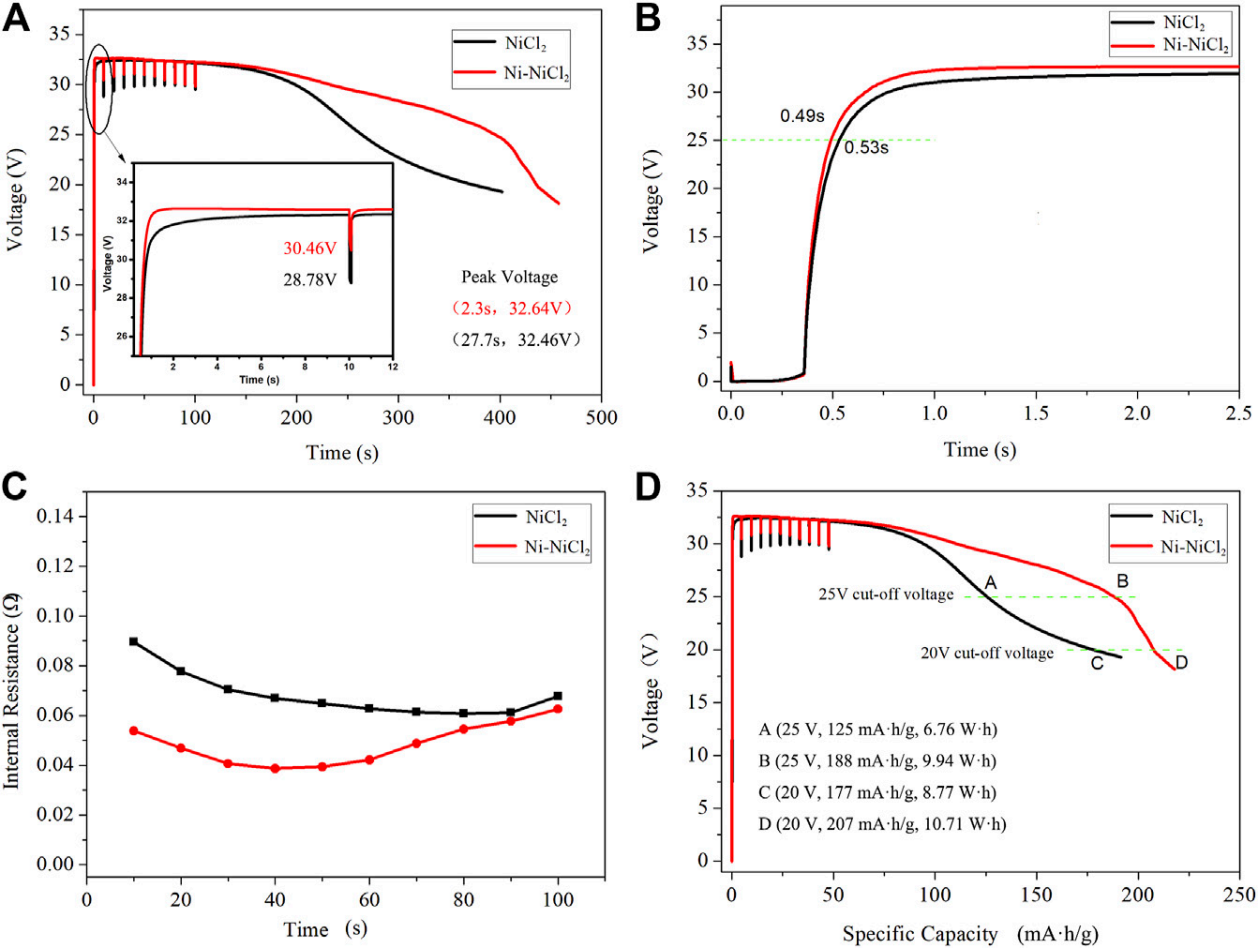
Discharge test results of thermal battery assembled using 13 cells at a constant current density of 100 mA/cm^2^ and the pulse current density (1 A/cm^2^, 10 ms) loaded every 10 s. **(A)** Voltage–time curve of NiCl_2_ and Ni-NiCl_2_, **(B)** the activation process, **(C)** voltage-specific capacity curves of NiCl_2_ and Ni-NiCl_2_, and the **(D)** internal resistance–time curves of NiCl_2_ and Ni-NiCl_2_.

The electrochemical performances of various cathode materials were subsequently investigated. The thermal battery based on the Ni-NiCl_2_ cathode possesses a longer working time and smaller pulse voltage drop compared to those of NiCl_2_ (Figure 5A). The voltage curves appear significantly different, and the voltage of Ni-NiCl_2_ is higher than that of NiCl_2_ (e.g., 29.25 vs. 25.03 V, respectively, at 263 s), the peak voltage (Supplementary Figure S4) also appears earlier than that of NiCl_2_ (e.g., 32.64 V at 2.3 s vs. 32.46 V at 27.7 s). Additionally, the curve corresponding to Ni-NiCl_2_ is above that of NiCl_2_ and ramps up quickly, demonstrating a shorter activation time. After loading the pulse current, the voltage of Ni-NiCl_2_ and NiCl_2_ is 30.46 and 28.78 V, respectively. Figure 5B indicates that the virtual voltage is ∼2 V prior to the activation, and Ni-NiCl_2_ outperforms NiCl_2_ in terms of the activation time (0.49 vs. 0.53 s). Essentially, when the test system inputs an electrical signal, the battery does not immediately output the current or voltage, because the combustion process of the firework system takes ∼0.3 s to start. Moreover, the voltages of the different batteries are observed to increase with different slopes. According to Ohm's law (the electromotive force *E= U + IR*_*i*_ or *U = E-IR*_*i*_), when the internal risistance *R*_*i*_ decreased, the output voltage *U* would increased. In this work, for untreated NiCl_2_ with a poor conductivity (Chu et al., 2016), the NiCl_2_-based thermal battery achieved the normal voltage only after the production of metallic Ni *via* the slow electrochemical reaction (Ni^2+^ + 2e → Ni) (Jin et al., 2017), as the metallic helped to decrease the internal resistance. However, for Ni-NiCl_2_, the already existing metallic Ni enables a significant enhancement in the conductivity and therefore contributed to rapidly decreasing the internal resistance and improving the output voltage. Therefore, the output voltage value would reach faster to activation voltage value for Ni-NiCl_2_ batteries. Figure 5C further demonstrates that Ni-NiCl_2_ shows a significantly lower internal resistance (0.054 vs. 0.090 Ω at 10 s), especially in the initial 60 s. Therefore, the activation time is successfully reduced owing to the reduced internal resistance. In addition, Figure 5D indicates that at a cut-off voltage of 25 V, the work time is 395 s and the specific capacity is ∼188 mAh/g for the Ni-NiCl_2_ thermal battery, whereas those of pure NiCl_2_ are 263 s and 125 mAh/g, respectively. The output energy (at the cut-off voltage of 25 V) of Ni-NiCl_2_ and NiCl_2_ are 9.94 and 6.76 Wh, respectively, which indicates an increase of 47% in the former. But with the cut-off voltage decrease?the performance gap is getting smaller and smaller?when the cut-off voltage is 20 V, the output energy of Ni-NiCl_2_ and NiCl_2_ are 10.71 and 8.77 Wh, respectively, which indicates an increase of 22% of the former composite material.

Several factors are possibly responsible for the enhanced performance of Ni-NiCl_2_. First, the Ni-NiCl_2_ composite was prepared *via* hydrogen reduction, which significantly reduced the direct pyrohydrolysis of NiCl_2_ during the discharging process. Therefore, the formation of the insulating nickel oxide was avoided, and crystal water was removed. Second, the formed metallic Ni reduced the internal resistance of thermal battery, thereby significantly accelerating the electrochemical reaction kinetics. Notably, the already existing Ni possibly served as active nucleation sites for the subsequent discharge process, which resulted in the thermal batteries rapidly achieving the working voltage. Finally, the battery was fabricated using a powder cold-press method; this cathode configuration could also significantly affect the final performance. The metallic Ni in Ni-NiCl_2_ can effectively inhibit the relative sliding of the materials, which facilitates the molding process for the cathode and avoids the conglutination of materials on the mold.

**FIGURE 6:**
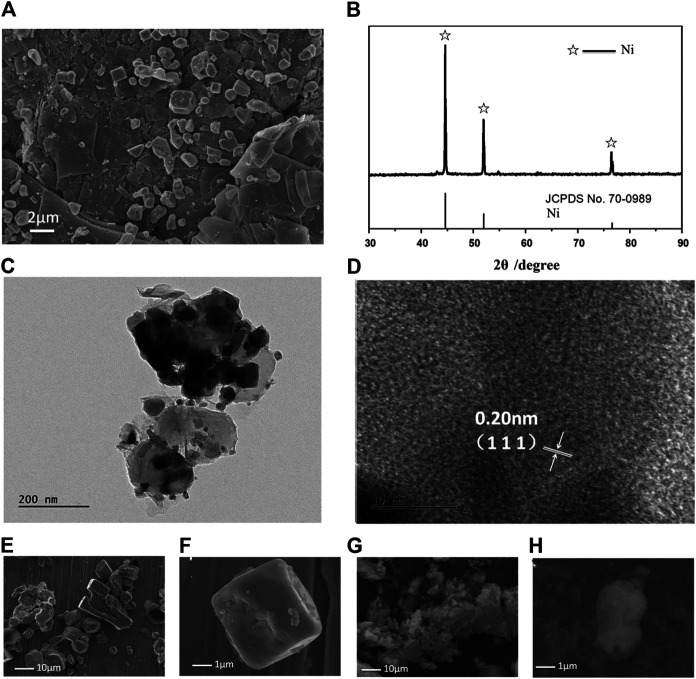
Evolution of product morphologies after the discharge tests. **(A)** SEM image, **(B)** XRD pattern, and **(C–D)** TEM and HRTEM images of the products. SEM images of the products are acquired in different discharge modes: **(E, F)** fast discharge mode (discharging current density of 1 A/cm^2^, 50 s to reach 0 V) and **(G, H)** no-load mode (maintaining an open-circuit until the battery is cold).

After the discharging process, the cathode product was dissolved in water, and a solid residue was obtained *via* magnetic separation. As shown in Figure 6A, numerous particles with different shapes attached to the layered sheet are observed in the SEM image. The XRD pattern (Figure 6B) reveals that the main phases correspond to metallic Ni (#70–0989 in ICSD patterns). Moreover, in Figures 6C,D, several Ni particles with a d-spacing of 0.203 nm (assigned to the (111) plane) are observed. Therefore, metallic Ni can be presumed to be the electrode reaction product. Furthermore, NiCl_2_ is known to possess a strong affinity with LiCl_2_ (Lemaire et al., 1997), implying that they can react with each other at a high temperature. During the experiments, the dissolution process was initiated only after a prolonged duration of over 60 s (Supplementary Figure S5). Considering the different morphologies of the discharged products, the product shape was thought to vary with an increase in the discharging duration. As shown in Figures 6A,E–H, rapid discharging (50 s) tends to form cubic particles, whereas floccules are formed upon no-load mode processing. Therefore, the different products shown in Figure 6A are presumed to be formed at different discharging times (Supplementary Figure S6). Extensive investigations on the manner in which the discharge process affects the formation of final products can be conducted in the future, which can provide a path for synthesizing particles with different shapes.

**FIGURE 7:**
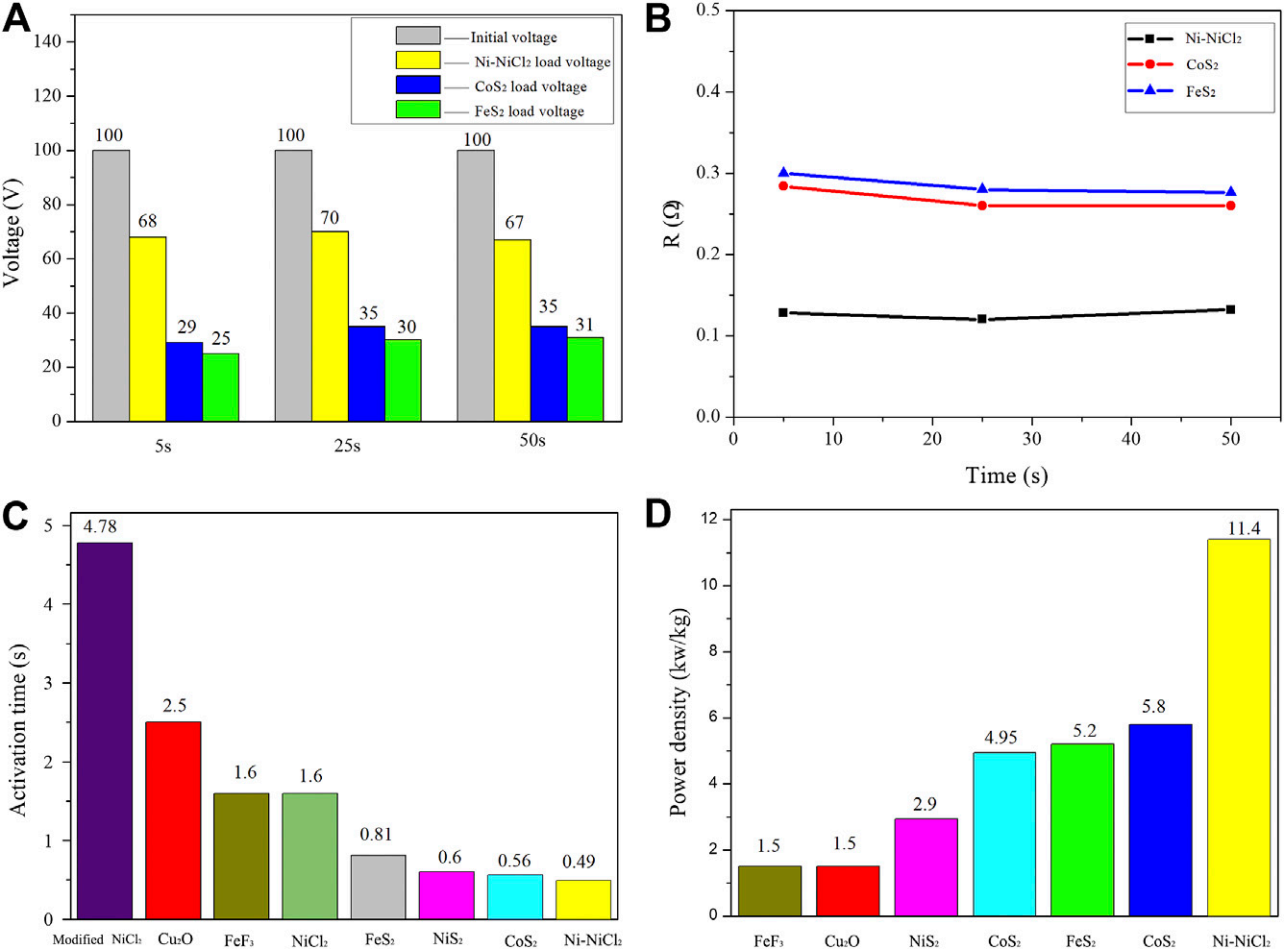
**(A)** Voltage drop and **(B)** internal resistance of 100 V thermal batteries after the loading of pulse current density (6 A/cm^2^, 10 ms) at 5, 20, and 50 s. **(C)** Activation time and **(D)** power density of different thermal batteries.

To further confirm the practical application-based performance of Ni-NiCl_2_, three 100 V thermal batteries were fabricated using FeS_2_ (natural pyrite), CoS_2_ (Hunan Ruilin New Energy Technology), and Ni-NiCl_2_. When the pulse current density (6 A/cm^2^) is loaded at 5, 20, and 50 s, the Ni-NiCl_2_ battery significantly outperforms sulfide-based batteries in terms of the power and internal resistance. For example, the voltage decreases from 100 to 70 V under a current density of 6 A/cm^2^ in the Ni-NiCl_2_ battery at 25 s. The voltage drop in the Ni-NiCl_2_ system (Figure 7A) is 30 V and the corresponding internal resistance is 0.12 Ω (Figure 7B), which is significantly lower than that of the FeS_2_ (70 V, 0.28 Ω) and CoS_2_ (65 V, 0.26 Ω) thermal batteries. Moreover, as shown in Figure 7C, the activation time of the Ni-NiCl_2_ battery is significantly lower than those of previously reported thermal batteries (Liu and Chu, 2004; Yu et al., 2018; Guo et al., 2019; Gui et al., 2020; Guo et al., 2020; Luo et al., 2020), which indicates the quick response ability of the battery. The power density of Ni-NiCl_2_ (11.4 kW/kg), as shown in Figure 7D, is considerably higher than those of several previously reported electrode materials (Zhu et al., 2012; Yu et al., 2018; Guo et al., 2019; Gui et al., 2020; Guo et al., 2020; Luo et al., 2020). In addition, the comparison of the power density of the present thermal batteries is summarized in Supplementary Table S2. These results further confirm the excellent performance of the battery.

## Conclusion

An Ni-NiCl_2_ composite was synthesized *via* the hydrogen reduction method to increase the power density and reduce the activation time of thermal batteries. As the reduced reaction product, metallic Ni grew *in situ* in the NiCl_2_ substrate and significantly improved the conductivity of the cathode material and in turn, the performance of the assembled thermal battery. The discharge tests revealed that the initial internal resistance considerably decreased and the activation time reduced. Notably, the Ni-NiCl_2_ composite cathode increased the output energy by ∼47% when the thermal batteries were used in weapons (working voltage >25 V), with a power density of up to 11.4 kW/kg. The improvement in the fast response ability of batteries is vital for emergency equipment and weapon systems. Therefore, NiCl_2_ was found to be an ideal material for replacing sulfide cathode materials in high-power thermal batteries. The exploration of compatible electrolyte materials and investigation of the growth mechanism of the Ni products can be considered in the future for the further development of thermal batteries.

## Data Availability

The original contributions presented in the study are included in the article/Supplementary Material, further inquiries can be directed to the corresponding author.
