# Supplementary material for: Fabrication of the Ni-NiCl2 Composite Cathode Material for Fast-Response Thermal Batteries
Source: Front Chem. 2021 May 17;9:679231. doi: 10.3389/fchem.2021.679231 (PMC8165393; doi:10.3389/fchem.2021.679231)
Supplement: Supplementary file 1 [file DataSheet1.docx]

Supplementary Material

Fabrication of the Ni-NiCl_2_ composite cathode material for fast-response thermal batteries

Qianqiu Tian^1^, Jiajun Wang^1^, Wendi Xiang^1^, Jun Zhao^1^, Hao Guo^2^, Jing Hu^3^, Xiaopeng Han^1^* and Wenbin Hu^1^

^1^ School of Materials Science and Engineering, Tianjin University, Tianjin 300071, China. ^2^ State Key Laboratory of Advanced Chemical Power Sources, Guizhou 563003, China. ^3^ Shandong Engineering Research Center of Green and High-value Marine Fine Chemical，Weifang University of Science and Technology, Shouguang 262700, China

Corresponding

E-mail: xphan@tju.edu.cn;


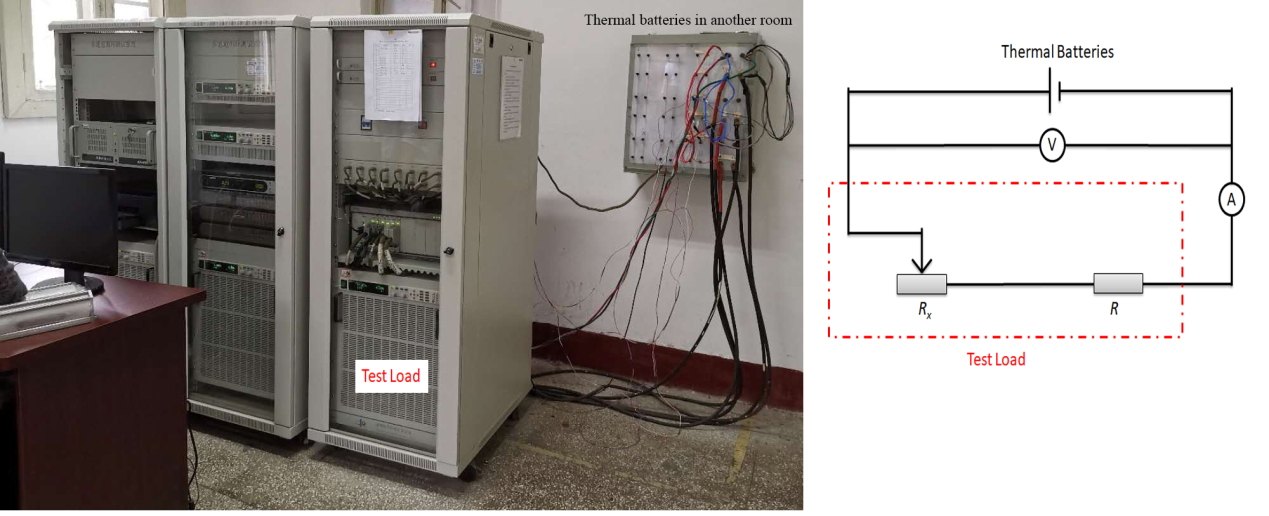


**Figure S1**. dedicated device for testing the thermal batteries.


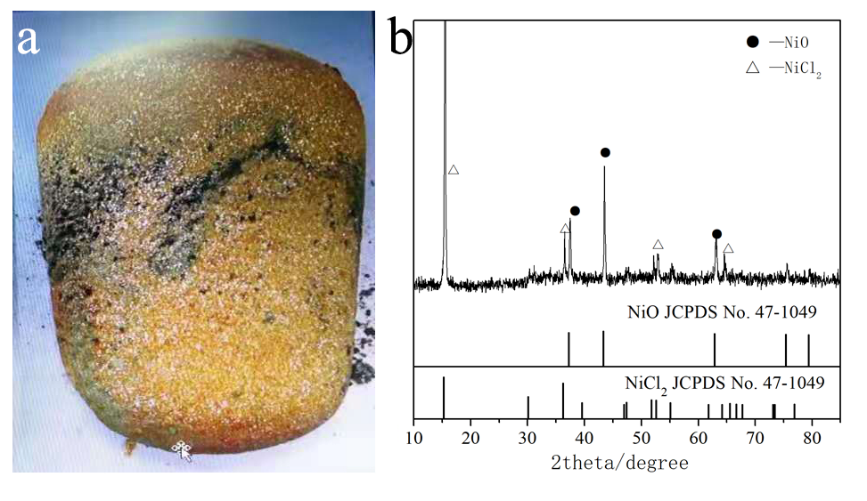


**Figure S2**. Roasted products of (a) The picture of NiCl_2_ contained NiO, (b) XRD pattern.


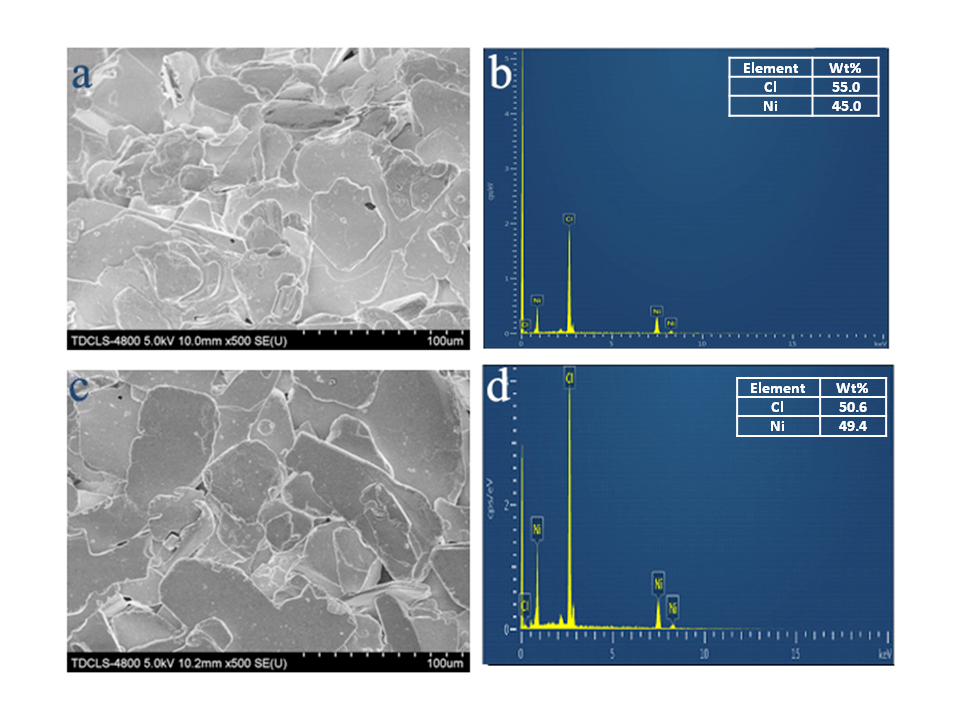


**Figure S3**. SEM and EDS of Roasted NiCl_2_ (a, b) and reduced NiCl_2_ (c, d).


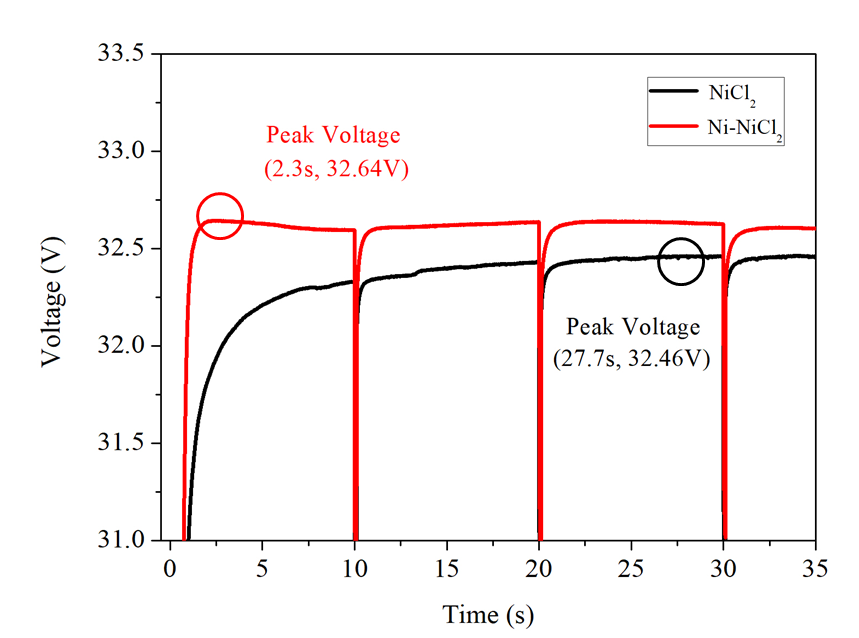


**Figure S4.** Peak voltage of thermal batteries based on two kinds of materials.


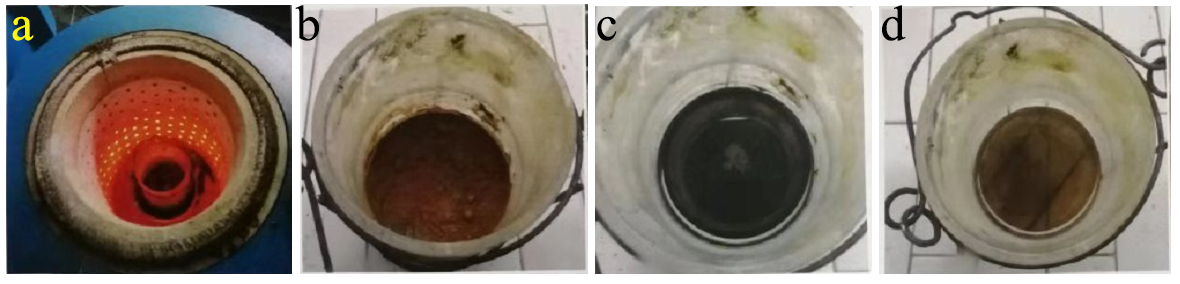


**Figure S5**. Photograph of heating oven (a), the mixture of NiCl_2_+LiCl after heating for 60s (b) and 180s (c), the LiCl after heating for 180s (d).


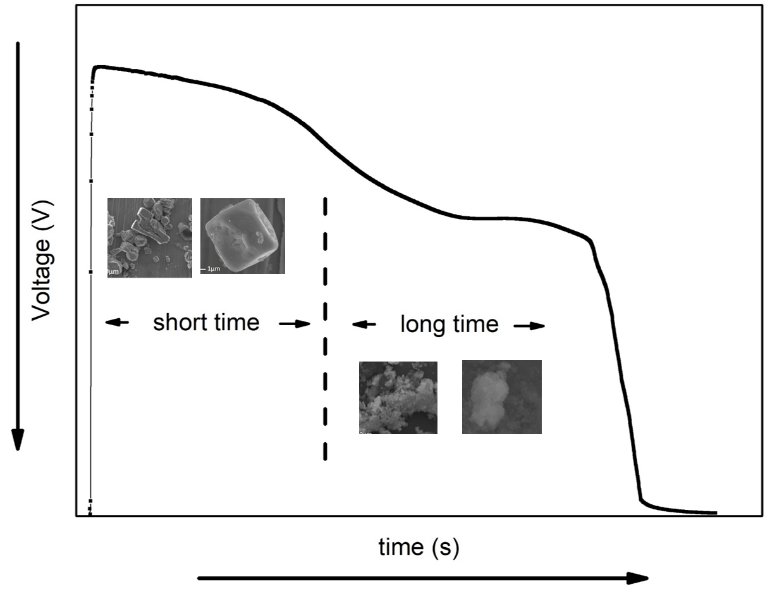


**Figure S6**. Illustration for the morphology change of products during the discharging process.

**Table S1** Proportion of materials

| **Component** | **Material** | **Proportion** | **Mass/g** |
| --- | --- | --- | --- |
| Anode | LiB alloy | 60%Li | 0.7 |
| Separator | MgO- Electrolyte | 50-50 | 3.5 |
| Cathode | NiCl_2_- Electrolyte-Carbon | 100-15-5 | 4.0 |
| Heating plate | Fe-KClO_4_ | 84-16 | 6.2 |
| Electrolyte | LiCl-LiF- LiBr | 9.6-22-68.4 | / |

**Table S2** Comparison of the power density of the present thermal batteries

| **Cathod material** | **Activation time/s** | **Power density/kw·kg^-1^** | **Reference** |
| --- | --- | --- | --- |
| Modified NiC_l2_ | 4.78 | / | Gui et al., 2020 |
| Cu_2_O | 2.5 | 1.5 | Luo et al., 2020 |
| FeF_3_ | 1.6 | 1.5 | Guo et al., 2019 |
| NiCl_2_ | 1.6 | / | Gui et al., 2020 |
| FeS_2_ | 0.81 | 5.2 | Yu et al., 2018 |
| NiS_2_ | 0.6 | 2.9 | Guo et al., 2020 |
| CoS_2_ | 0.56 | 4.95 | Liu et al., 2004 |
| Ni-NiCl_2_ | 0.49 | 11.4 | Our work |

# References

Gui, Y.F., Lin, X.X., Fu, L.C., Zhu, J.J., Yang, W.L., Li, D.Y., et al. (2020). Shortening activation time of thermal battery by hydrogen etching of NiCl2 cathode. Mater. Lett. 275, 128136. doi: 10.1016/j.matlet.2020.128136

Luo, Z.S.J., Fu, L.C., Zhu, J.J., Yang, W.L., Li, D.Y., and Zhou, L.P. (2020). Cu2O as a promising cathode with high specific capacity for thermal battery. J. Power Sources 448, 227569. doi: 0.1016/j.jpowsour.2019.227569.

Guo, S.N., Guo, H., Wang, X.Y., Zhu, Y.P., Hu, J., Yang, M., et al. (2019). Iron Trifluoride as a High Voltage Cathode Material for Thermal Batteries. J. Electrochem. Soc. 166(15), A3599-A3605. doi: 10.1149/2.0371915jes.

Yu F.S., Bai Y.X., Wang J.P., Zhao Y.L., and Kang E.W. (2018). Study on factors for activation time of thermal battery. Chinese J. Power Sources 42(12), 1885-1888. doi: 1002-087 X (2018)12-1885-04

Guo, H., Tang, L.C., Tian, Q.Q., Chu, Y., Shi, B., Yin, X.C., et al. (2020). Cobalt-Doped NiS_2_ Micro/Nanostructures with Complete Solid Solubility as High-Performance Cathode Materials for Actual High-Specific-Energy Thermal Batteries. ACS Appl. Mater. Inter. 12, 50377-50387. doi: 10.1021/acsami.0c13396.

Liu Y.D., and Chu D.W. (2004). Electrochemical characteristics of the LiSi-CoS_2_ thermal batteries. Chinese J. of Power Sources 28(11),693-696. doi: 1002-087 X(2004)11-0693-04.
